# *De novo* hydroponics system efficiency for the cuttings of alfalfa (*Medicago sativa* L.)

**DOI:** 10.1007/s12298-021-00995-3

**Published:** 2021-05-19

**Authors:** Zhili Zhao, Wenyu Zhang, Yang Liu, Shuai Li, Wu Yao, Xiaohui Sun, Siyu Li, Lichao Ma, Juan Sun, Qingchuan Yang, Yongxiang Li, Guofeng Yang, Zeng-Yu Wang, Lili Cong

**Affiliations:** 1 College of Agronomy, Hunan Agricultural University, Changsha, 410128 China; 2 College of Grassland Science, Grassland Agri-Husbandry Research Center, Qingdao Agricultural University, Qingdao, 266109 China; 3 Institute of Animal Sciences, Chinese Academy of Agricultural Science, Beijing, 100193 China; 4 Qingdao Empyrean Intelligent Agriculture Group Co, Ltd, Qingdao, 266109 China

**Keywords:** Alfalfa, Hydroponic, Cutting propagation, Rooting rate

## Abstract

The legume plant alfalfa (*Medicago sativa* L.) is a widely cultivated perennial forage due to its high protein content, palatability, and strong adaptability to diverse agro-ecological zones. Alfalfa is a self-incompatible cross-pollinated autotetraploid species with tetrasomic inheritance. Therefore, maintaining excellent traits through seed reproduction is a prime challenge in alfalfa. However, the cutting propagation technology could enable consistent multiplication of quality plants that are genetically identical to the parent plant. The current study aimed to develop a simple, cost-effective, reproducible, and efficient hydroponic cutting method to preserve alfalfa plants and for molecular research. In this study, alfalfa landrace ‘Wudi’ was grown in hydroponics for 30 days and used as source material for cuttings. The top, middle and bottom sections of its stem were used as cuttings. The rooting rate, root length, and stem height of the different stem sections were compared to determine the best segment for alfalfa propagation in four nutrient treatments (H^M^, H^M^ + 1/500H, H^M^ + 1/1000H and d H^M^ + 1/2000H). After 21 days of culture, the rooting rates of all the three stem types under four cutting nutrient solutions were above 78%. The rooting rate of the middle and bottom parts in H^M^ + 1/1000 H and H^M^ + 1/2000 H nutrient solutions reached more than 93%, with a higher health survey score (> 4.70). In conclusion, this study developed a de novo cutting propagation method that can be used to conserve and propagate germplasm in breeding programs and research. This method is a new report on the cutting propagation of alfalfa by hydroponics, which could supplement the existing cutting propagation methods.

## Introduction

Alfalfa (*Medicago sativa* L.), known as the “Queen of forage”, is the most important leguminous grass worldwide, with the advantages of high nutritional value, palatability, high-stress resistance, and nitrogen-fixing ability (Shen et al. 2020; Ma et al. 2020). Rapid increase in livestock production has also increased the demands for alfalfa forage across the world (Bai et al. 2018). Besides, its rich genetic diversity enables its cultivation under various environmental conditions. Therefore, alfalfa is not only a high-quality feedstock but also important for the ecological conservation (Radović et al. 2009).

The recent transformation and upgradation of animal husbandry has increased the demand for alfalfa cultivars in farmers. However, cultivated alfalfa species has a self-incompatible cross-pollinated autotetraploid system (2n = 4 × 8 = 32) with tetrasomic inheritance in which bivalent pairing is not favorable during hybridization (McCoy and Bingham 1988; Chen et al. 2020). Therefore, the maintenance of economically important traits through seed reproduction in alfalfa is a prime challenge for seed producing agencies. However, de novo cutting propagation method could enable consistent multiplication of plants that are genetically identical to the parents.

With the rapid advancement of modern research including genome editing technology, the molecular studies of plants have become more precise and in-depth to develop new cultivars. Therefore, consistent genetic purity of genotypes is required to avoid background interference of experimental results. Alfalfa with 25–75% natural outcrossing rate results in the same variety producing contaminated seeds may the breeding lines. Previous studies (Cong et al. 2017a, b; Cui et al. 2020; Kang et al. 2020; Xiong et al. 2020) on alfalfa proteomics, transcriptomics, and gene mining experiments used variety as source material with diverse genetic backgrounds, and such a mixed genotype will affect the accuracy of bioinformatics analysis and gene mining. However, the primary benefit of cuttings is that all offspring are genetically/ phenotypically similar clones as like maternal source.

Hydroponic cutting propagation is a form of plant propagation in which cuttings are multiplied in water and nutrient solution as a medium instead of soil. This method is advantageous because of the short cycle, rapid screening, and year-round production in a controlled environment. Plants grown under hydroponics systems typically produce higher yields, require less space, and conserve soil and water. This system is ideal when outdoor gardening space is limited (Wang et al. 2020). Moreover, the systems enable easier control of key factors in the growth environment, including temperature, light intensity, and moisture, which is conducive to maintain consistent quality of plant materials for research (Lee et al. 2020). Meanwhile, it also has the advantage of conveniently observing the entire root growth process (Delden et al. 2020).

Therefore, our objective was to develop a hydroponic cutting propagation system in alfalfa, which can simple, time-saving, and has a high propagation coefficient for clone preservation. The method can provide genetically identical alfalfa clones for scientific research, conventional alfalfa breeding, and molecular studies.

## Materials and methods

### Preparation of cutting materials

The ‘Wudi’ alfalfa landrace was selected as the parent plant for stock material, the seeds used are collected and preserved in our laboratory. Cutting materials preparation refer to the method as described by Cong et al. (2017a, b). Alfalfa seeds were sterilized in 70% ethanol for 1 min and rinsed in sterilized water five times. The seeds were placed in Petri dishes containing two sheets of sterile filter papers and maintained in a growth chamber under controlled conditions (25 ± 1 °C day/20 ± 1 °C night, 80% relative humidity, 16 h light/8 h dark). Seven days after germination, uniform seedlings were transferred into specially designed pots containing modified Hoagland nutrient solution (Table 1) and incubated under the following conditions: 16 h light (25 °C)/8 h darkness (20 °C), the light intensity of 300 mol/m^2^·s, and relative humidity of 75%. The nutrient solution was refreshed every seven days.

**Table 1:** Preparation of individual nutrient solutions for the culturing of alfalfa cuttings

Stock solution	Composition	Amount
A solution (200 ×)	Ca (NO_3_)_2_·4H_2_O	189.00 g
	KNO_3_	121.4 g
B solution (200 ×)	NH_4_H_2_PO_4_	23.0 g
	MgSO_4_·7H_2_O	98.6 g
C solution (1000 ×)	H_3_BO_3_	2.86 g
	MnSO_4_·4H_2_O	2.13 g
	ZnSO_4_·7H_2_O	0.22 g
	CuSO_4_·5H_2_O	0.08 g
	(NH4)_4_Mo_7_O_24_·4H_2_O	0.02 g
D solution (200 ×)	FeSO_4_·7H_2_O	5.561 g
	EDTA-Na_2_·2H_2_O	7.485 g
	ddH_2_O	To 1 L

While preparing the nutrient solution, each solute was dissolved separately and then mixed. The prepared stock solutions should be protected from light during storage. For 1 L 1/2 Hoagland nutrient solution, 2.5 mL stock solution A, B, and D and 0.5 mL solution C were added separately into 1 L distilled water

### Design of culture containers

**Fig. 1:**
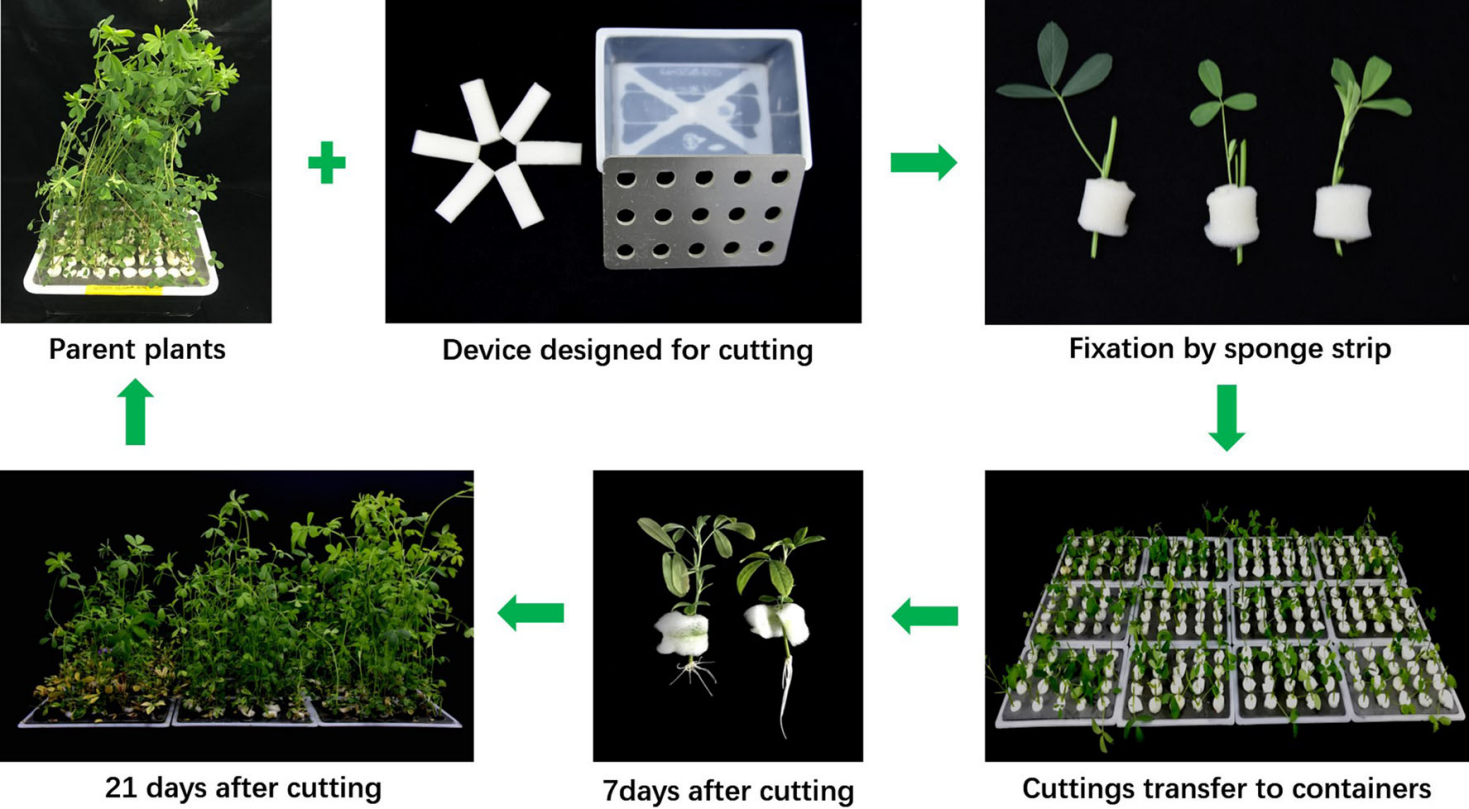
Schematic overview of alfalfa cutting in the hydroponic system

Design of the culture containers was modified base on the method as described by Cong et al. (2017a, b). As shown in Fig. 1, the culture containers consisted of white plastic pots (17.5 cm long, 11.5 cm wide, and 5 cm high). The outer surfaces of the culture containers were covered by a black tape to maintain a dark environment to promote root growth and prevent the growth of green algae in the nutrient solution. Further, the pots were covered with polyvinyl chloride (PVC) board, which was perforated with 15 evenly spaced holes (1.2 cm diameter) (Fig. 1a). The cut sponge strips (1 cm thick, 5–6 cm long and 1.8–2.0 cm wide) were washed 1–2 times with tap water and then washed once with distilled water. The culture pots, PVC boards, and the sponge strips were washed with 75% alcohol and kept in an ultra-clean platform for 4 h UV disinfection. PVC board can be reused many times and is easy to sterilize.

### Preparation of nutrient solutions

As shown in Tables 1 and 2, different concentrations of HB-101 natural plant nutrient solutions (v/v, 1/500, 1/1000, and 1/2000) were added to 1/2 modified Hoagland solution, respectively, and stored at room temperature until used for cultivation of cuttings.

**Table 2:** Preparation of culture medium for alfalfa cuttings

Composition	Volume
A solution	2.5 mL
B solution	2.5 mL
C solution	0.5 mL
D solution	2.5 mL
HB-101	2 mL (1/500);1 mL (1/1000);0.5 mL (1/2000)
ddH_2_O	To 1 L

The preparation protocols for solutions A, B, and C are shown in this table

### Cutting method

After 30 days of hydroponics culture, branches with a stem base diameter of 1.6–1.8 mm from healthy, disease-free plants were selected as source material for propagation cuttings. Each branch was divided into three segments: Top part (the top of the branch), middle part (the fifth stem segment), and the bottom part (the first stem segment). Stem sections of size 6–8 cm with 1–2 leaves and one bud were sliced at a 45-degree angle. While cutting the stems, it was ensured that the cuttings were not too large because big cuttings would not root well or, if rooted, the plants would be tall and lanky instead of being compact. Plant cuttings were transplanted into pots containing 750 mL of cutting nutrient solution and placed in position with a 1 × 3 cm sponge strip (~ 1 cm thick). All jars and sponge strips were sterilized before use with 75% ethanol for 2 h and then placed under ultraviolet light in a laminar flow hood for another 2 h. Each treatment contained 20 cuttings and experiment was done in 3 replications. Preparation of cuttings was done in the early morning between 7 and 8 am when the plant cells were turgid since the stems were easier to cut with a higher chances of maintaining turgidity after culture.

### Post-cutting management

In the first week after cutting, the pots were placed in a chamber under controlled conditions of temperature (25 ± 1 °C day/20 ± 1 °C night), humidity (80%), and photoperiod (16 h light (200 mol/m^2^ s)/8 h dark). After one week, the culture conditions were changed to 300 mol/m^2^·s light intensity, 16 h (25 °C) day/8 h night (20 °C), 75% relative humidity. The nutrient solution was refreshed every seven days. The plant growth parameters of five cuttings per treatment were assessed 21 days after culture, including the percentage of rooted cuttings, root length, and stem height. The five rooted plants per treatment were used for the health survey on a scale of 1–5, where: 1–2 scores = Plant was severely stunted, leaves discolored or the plant completely dead; 2–3 scores = Plant grows weakly, roots and leaves discolored, root length is short (< 5 cm); 3–4 scores = Plant grows well, root system developed; 4–5 scores = Plant has more lateral roots, plant grow luxuriant. Rooted alfalfa cuttings were subsequently cultivated for another seven days and used for experiments or planted in pots. The young plants needed special care to ensure optimal growth.

### Data analysis

Analyses of variance (one-way ANOVA) were conducted using SPSS software version 19.0 (SPSS Inc., Chicago, IL, USA) for significant differences among treatments and to analyze the influence of different stem parts and various nutrient solutions on the rooting rate, root length and stem height. Means were separated using Duncan’s multiple range test at *P* < 0.05).

## Results

### The bottom segment showed the best efficiency in cutting propagation

**Fig. 2:**
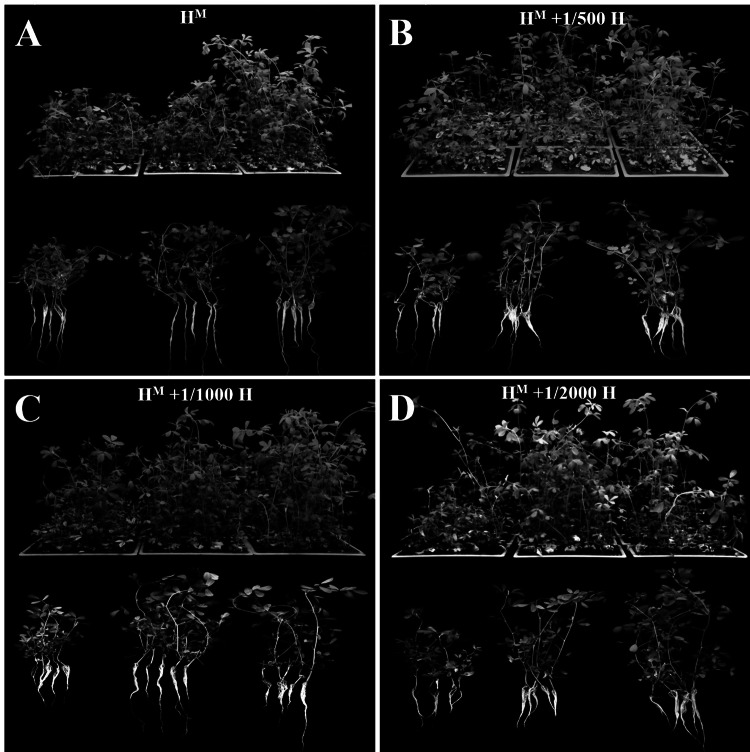
Response of different stem parts on the propagation of cuttings under four nutrient treatments: **A** H^M^ nutrient solution. **B** H^M^ + 1/500H nutrient solution. **C** H^M^ + 1/1000H nutrient solution. **D** H^M^ + 1/2000H nutrient solution

In this study, rooting occurred 7 days after culture. The data presented in Fig. 2A–D indicates that cuttings were well rooted after 21 days of culture. The rooting rates of all the three stem types under four cutting nutrient treatments were above 78%, indicating that the new cutting rooting method developed in this experiment has an excellent rooting efficiency (Table 3).

**Table 3:** Effect of different stem parts on cutting propagation

Stem part for cutting	Nutrient solution	Rooting rate (%)	Root length (cm)	Stem height (cm)	Health survey score
Top	H^M^	83.33 ± 1.67a	9.99 ± 0.55a	9.51 ± 0.47b	3.44 ± 0.14b
Middle	H^M^	86.67 ± 6.01a	13.37 ± 2.13a	14.61 ± 3.29ab	3.74 ± 0.13b
Bottom	H^M^	91.67 ± 1.67a	9.33 ± 0.59a	18.74 ± 1.48a	4.43 ± 0.12a
Top	H^M^ + 1/500 H	83.33 ± 1.67a	6.86 ± 0.50b	8.36 ± 0.57b	2.92 ± 0.14c
Middle	H^M^ + 1/500 H	93.33 ± 1.67a	7.80 ± 0.17ab	15.87 ± 2.53a	3.36 ± 0.09b
Bottom	H^M^ + 1/500 H	93.33 ± 4.41a	8.58 ± 0.20a	19.18 ± 1.0a	4.60 ± 0.13a
Top	H^M^ + 1/1000 H	78.33 ± 6.01b	7.37 ± 0.25b	8.47 ± 0.68c	3.34 ± 0.11b
Middle	H^M^ + 1/1000 H	93.33 ± 3.33a	8.93 ± 0.06b	15.09 ± 1.62b	4.72 ± 0.09a
Bottom	H^M^ + 1/1000 H	98.33 ± 1.67a	12.72 ± 1.70a	19.45 ± 0.27a	4.88 ± 0.04a
Top	H^M^ + 1/2000 H	90.00 ± 0.00b	7.85 ± 0.79b	10.09 ± 1.30b	3.10 ± 0.16b
Middle	H^M^ + 1/2000 H	100.00 ± 0.00a	9.00 ± 1.14ab	16.70 ± 2.69ab	4.86 ± 0.05a
Bottom	H^M^ + 1/2000 H	98.33 ± 1.67a	10.94 ± 0.50a	20.95 ± 2.39a	4.76 ± 0.09a

The data is shown as the mean ± SE (*n* = 3), and different letters at the top of each column indicate significant differences under the same nutrient solution (*P* < 0.05)

The material of cuttings is an important limiting factor for the survival of cuttings. There were significant differences in the rooting rates, root length, stem height between the top, middle, and bottom segments. Under the four nutrient treatments (H^M^, H^M^ + 1/500H, H^M^ + 1/1000H, H^M^ + 1/2000 H), rooting rates of the top segment were observed with 83.33%, 83.33%, 78.33%, 90.00% rooting rate respectively. The middle segments (86.67%, 93.33%, 93.33%, 100%) and bottom segments (91.67%, 93.33%, 98.33%, 98.33%) showed better rooting efficiency under H^M^, H^M^ + 1/500H, H^M^ + 1/1000H, H^M^ + 1/2000H nutrient treatments. Moreover, rooting lengths, stem heights of bottom segment were higher than other parts. The highest health survey score was achieved in the bottom segment (4.43, 4.60, 4.88, 4.76), followed by the middle segment (3.74, 3.36, 4.72, 4.86) and top segment (3.44, 2.92, 3.44, 3.10) under H^M^, H^M^ + 1/500H, H^M^ + 1/1000H, H^M^ + 1/2000H nutrient treatments. In conclusion, the bottom segment was the ideal choice for cutting propagation in this study, and all cuttings grow well with healthy root system and leaves (Fig. 2C, D).

### H^M^ + 1/2000H nutrient treatment is the optimal option in this new cutting propagation method

**Fig. 3:**
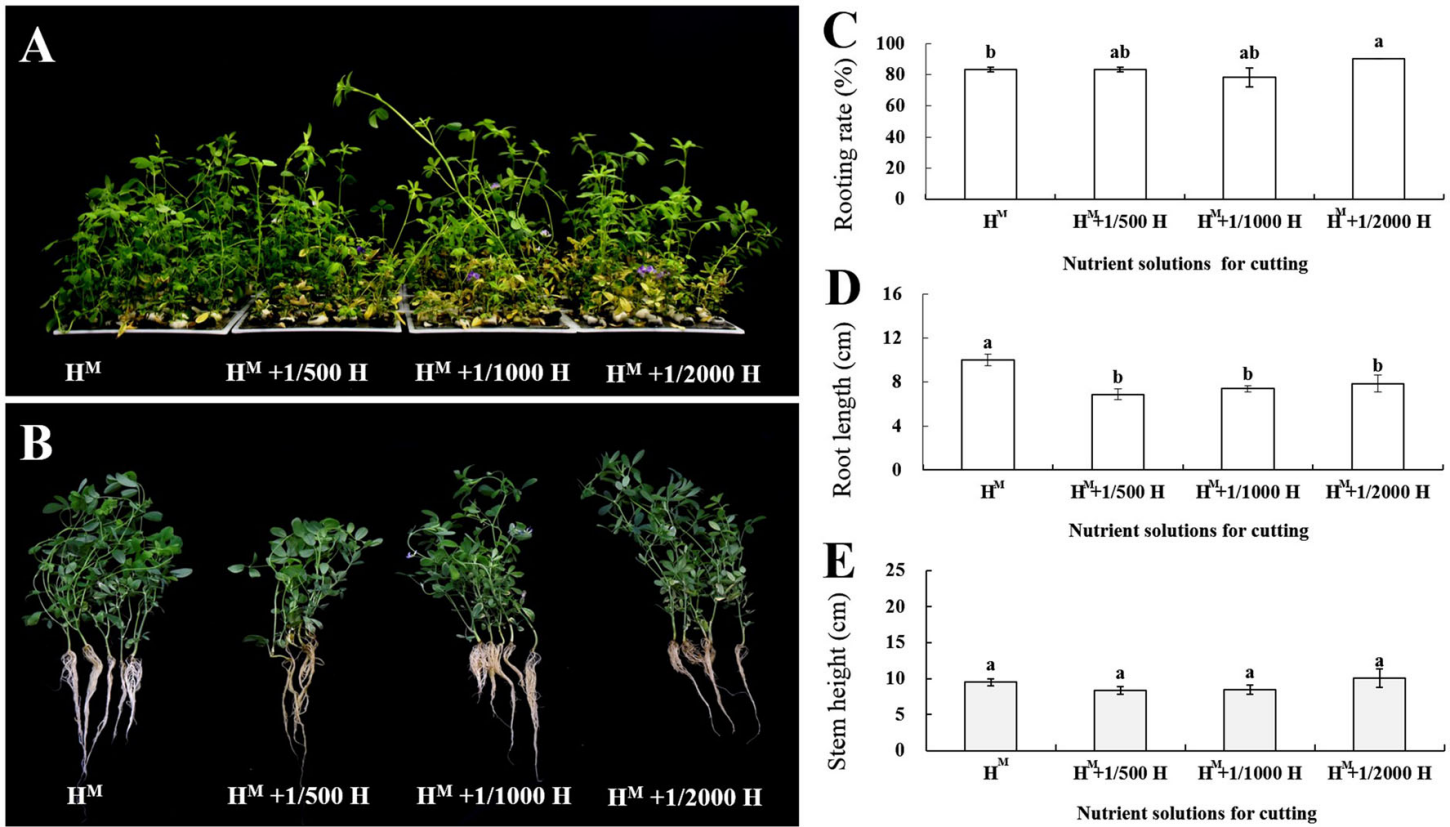
Rooting rates, root lengths, and stem heights of the top stem segment obtained in the four nutrient treatments: **A** cuttings grown in the four nutrient treatments. **B** Rooted plants in the four nutrient treatments. **C** Rooting rate. **D** Root length. **E** Stem height

After 21 days of culturing, Table 2 shows that different nutrient treatments have significant effects on the rooting rate, root length, stem height, and health survey score (*P* < 0.05). For the top stem segment, the highest rooting rate was observed in H^M^ + 1/2000H (90.00%), while there were no significant differences in the rooting rates of the other three nutrient solutions (83.33%, 83.33%, and 78.33% for H^M^, H^M^ + 1/500H, and H^M^ + 1/1000H, respectively). However, H^M^ led to greater root length (9.99 cm), but no significant differences were found in the other three nutrient treatment (Fig. 3). In terms of the stem height of the cuttings, H^M^ + 1/2000H attains the best rooting rates (average of 10.09 cm), followed by H^M^, H^M^ + 1/500H, and H^M^ + 1/1000H, respectively, but with no significant differences.

**Fig. 4:**
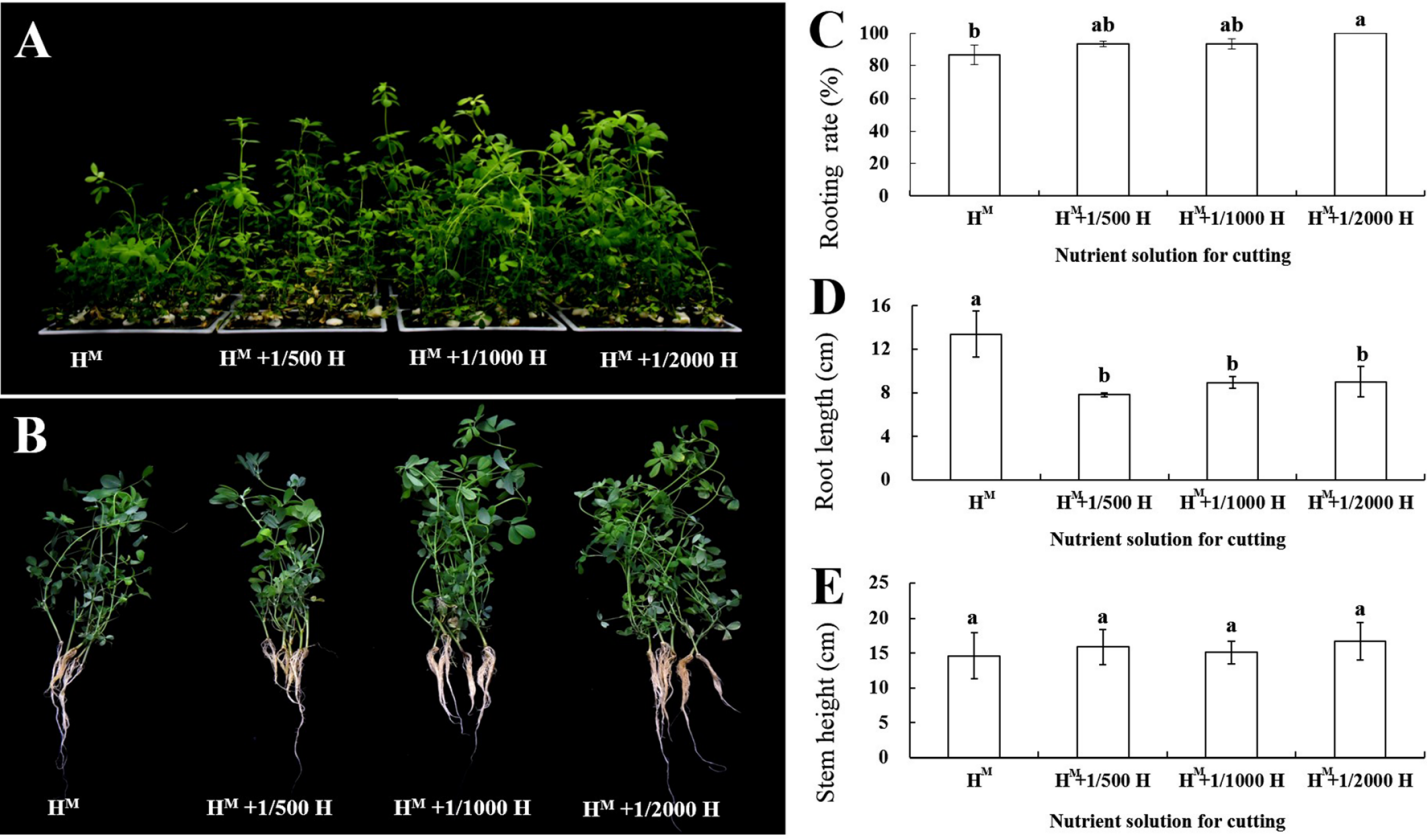
Rooting rates, root lengths, and stem heights for the middle stem segment under the four nutrient treatments: **A** cuttings grown in the four nutrient treatments. **B** Rooted plants in the four nutrient treatments. **C** Rooting rate. **D** Root length. **E** Stem height

Meanwhile, the rooting rates of the middle segment in the H^M^ + 1/2000H nutrient treatment reached 100%, and all the cuttings rooted well. The lowest rooting rates (86.67%) and the longest root length (13.37 cm) were found in the H^M^ nutrient solution. There were no significant differences in the stem height among the four nutrient solutions, but the stem height could reach more than 14 cm (Fig. 4).

**Fig. 5:**
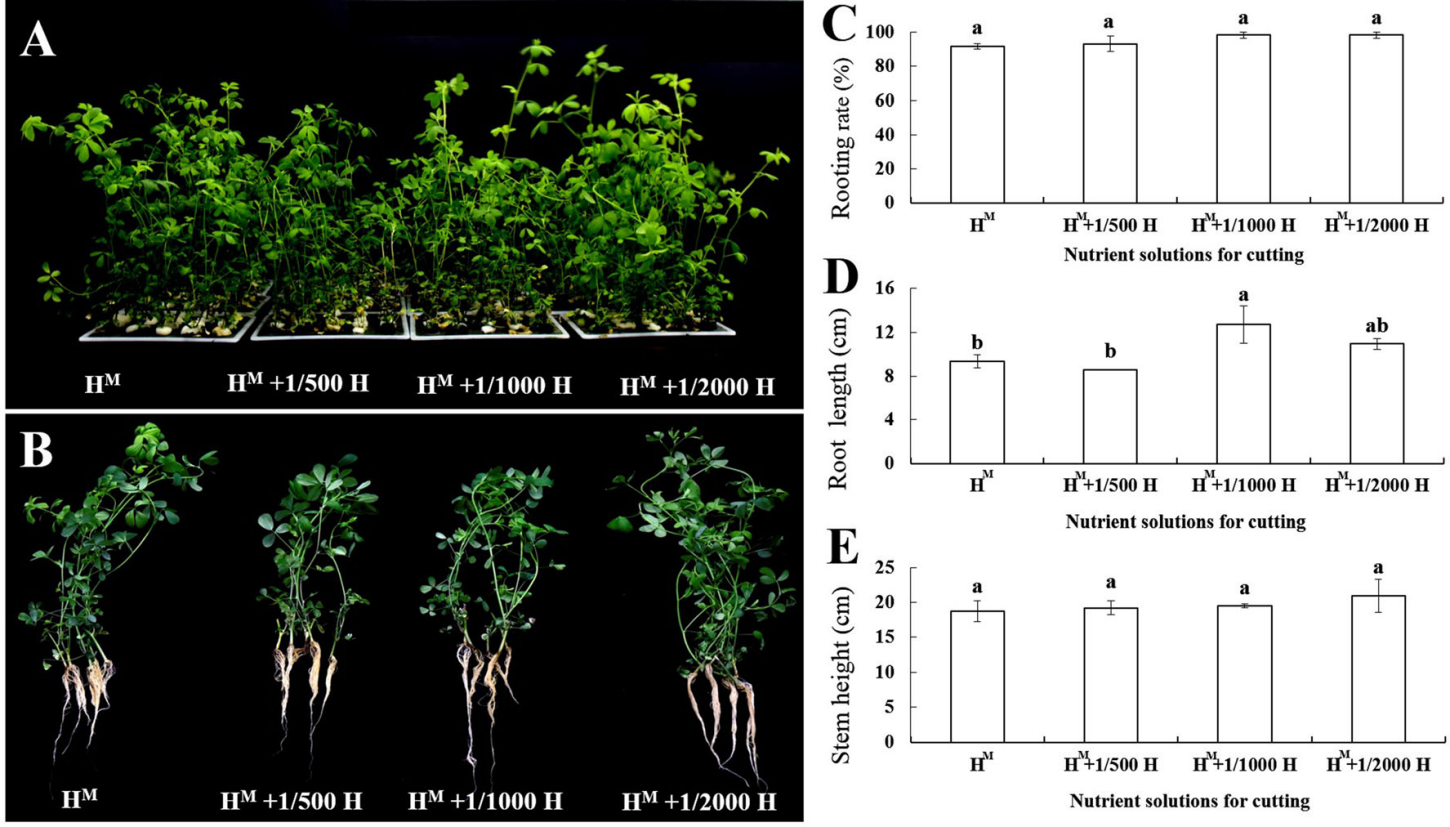
Rooting rates, root lengths, and stem heights for the lower stem segment grown under the four cutting nutrient treatments: **A** cuttings grown in four nutrient treatments. **B** Rooted plants in the four nutrient treatments. **C** Rooting rate. **D** Root length. **E** Stem height

As shown in Fig. 5 the rooting rate of the bottom stem segment showed no significant difference among the four cutting nutrient solutions, and all of which were higher than 90.00%. The highest rooting rate was observed in H^M^ + 1/2000H and H^M^ + 1/1000 H (98.33%), followed by H^M^ + 1/500H (93.33%), and H^M^ obtained the lowest (91.67%). The root growth was best in H^M^ + 1/1000H, with the length of the root reaching 12.72 cm, followed by H^M^ + 1/2000H (10.94 cm) and H^M^ (9.33 cm). Root growth was lowest in H^M^ + 1/500H, in which the root length (8.58 cm) was 4.14 cm shorter than in H^M^ + 1/1000H. According to the above data, H^M^ + 1/2000H nutrient treatment has a positive effect on root induction, which can be used in alfalfa cutting propagation.

## Discussion

At present, the commonly used cutting methods in alfalfa include field cuttings and indoor seedling cuttings, both of which are soil-based. Although the survival rate of some germplasm by these methods can reach 85%, the growth consistency of plants generated by the above methods is suboptimal, and thus unsuitable for molecular studies as they may cause background results interference. Therefore, establishing hydroponic systems for cutting propagation could solve these challenges.

Hydroponic methods have become more popular because they are space saving, dirt-free, and generate genetically consistent cutting plants within a short time (Wang et al. 2020). We demonstrated that the rooting rate of alfalfa cuttings is influenced by many factors, including the cuttings and culture mediums. Although all the stem segments in this study showed higher rooting rates than other methods reported previously (Niu et al. 2007), it was evident that the rooting ability of the bottom stem segment is the optimal choice for cutting propagation under the hydroponics system, followed by the middle part and top part. It is possible that the bottom and middle stem segments are fully developed and can provide the primary nutrients for rooting and initial growth of cuttings. In contrast, the developmental stage of the top stems is relatively young, and the inadequate storage of nutrients is not conducive for rooting (Hackett 1988; Niu et al. 2007; Haissing and Riemenschneider 1992). A similar study about alfalfa cutting in soil also demonstrated that bottom and middle stem segments have better rooting capacity, but the middle segment obtain the highest rooting rate (85.4%). The possible reason for this result is the different growth period of the cuttings.

In our exploratory experiment, we found that the composition of the nutrient solution was very important for stimulating the rooting rate under the hydroponics system. We tried to use Hoagland’s nutrient solution for root formation, but failed to induce rooting. With several attempts, we found that three stem segment types can root well when cultured in 1/2 Hoagland nutrient solution, and the effect enhanced when 1/2000 (V/V) HB-101 natural plant nutrient solution was added. This result suggests that low-nutrient may be beneficial to the growth of root. Previous studies have reported that plants under nitrogen and phosphorus limitation increase resource allocation to root growth relative to shoot growth (Schneider et al. 2021; Liu 2021). To cope with nitrogen and phosphorus deficiency, a typical response is the inhibition of photosynthesis and an increase in the root/shoot ratio due to a decrease in shoot growth and to an increase in the allocation of carbon from shoots to roots (Hermans et al. 2006; López-Arredondo et al. 2014; Schneider et al. 2021). In addition, our results showed that HB-101 supplementation at concentrations of 1/1000 and 1/2000 could stimulate the growth of roots. However, the rooting rate in H^M^ + 1/500H was higher than in H^M^, but H^M^ + 1/500 H limited root length in all the three stem segments. It is possible that a low concentration of HB-101 can promote rooting but could inhibit root growth when used at higher concentrations.

## Conclusions

In conclusion, we developed a convenient and efficient method for producing clones with high genetic consistency by the cutting method in alfalfa. Under H^M^ + 1/2000H nutrient solutions, rooting rate can be reached 98.33% using the basal segment, and cuttings developed a better root system. The optimized technique is feasible for cutting propagation under the hydroponics system for alfalfa and can be conducted at any time during the active growth period of parent plants. Moreover, the technique unlocked the bottlenecks experienced in soil propagation of alfalfa cuttings, making it easier to provide large quantities of cloning materials with growth consistency for molecular research applications such as genomic, transcriptomic, and proteomic analyses. This de novo cutting method for alfalfa has the potential for application in conventional breeding and molecular studies.

## Abbreviations

H^M^: Modified 1/2 Hoagland nutrient treatment
H^M^ +1/500H: 1/500(v/v) HB-101 added to modified 1/2 Hoagland nutrient treatment
H^M^ +1/1000H: 1/1000(v/v) HB-101 added to modified 1/2 Hoagland nutrient treatment
H^M^ +1/2000H: 1/2000(v/v) HB-101 added to modified 1/2 Hoagland nutrient treatment
